# Polymorphisms in Genes Involved in the NF-κB Signalling Pathway Are Associated with Bone Mineral Density, Geometry and Turnover in Men

**DOI:** 10.1371/journal.pone.0028031

**Published:** 2011-11-21

**Authors:** Delnaz Roshandel, Wendy Thomson, Stephen R. Pye, Steven Boonen, Herman Borghs, Dirk Vanderschueren, Ilpo T. Huhtaniemi, Judith E. Adams, Kate A. Ward, Gyorgy Bartfai, Felipe F. Casanueva, Joseph D. Finn, Gianni Forti, Aleksander Giwercman, Thang S. Han, Krzysztof Kula, Michael E. Lean, Neil Pendleton, Margus Punab, Frederick C. Wu, Kate L. Holliday, Terence W. O'Neill, the EMAS Study Group

**Affiliations:** 1 Arthritis Research UK Epidemiology Unit, The University of Manchester, Manchester Academic Health Science Centre, Manchester, United Kingdom; 2 Leuven University Division of Geriatric Medicine, Katholieke Universiteit Leuven, Leuven, Belgium; 3 Department of Andrology and Endocrinology, Katholieke Universiteit Leuven, Leuven, Belgium; 4 Department of Surgery and Cancer, Imperial College London, Hammersmith Campus, London, United Kingdom; 5 Clinical Radiology, Imaging Science and Biomedical Engineering, The University of Manchester, Manchester Academic Health Science Centre, Manchester, United Kingdom; 6 MRC-Human Nutrition Research, Elsie Widdowson Laboratory, Cambridge, United Kingdom; 7 Department of Obstetrics, Gynaecology and Andrology, Albert Szent-Gyorgy Medical University, Szeged, Hungary; 8 Department of Medicine, Santiago de Compostela University, Complejo Hospitalario Universitario de Santiago (CHUS), CIBER de Fisiopatología Obesidad y Nutricion (CB06/03), Instituto Salud Carlos III, Santiago de Compostela, Spain; 9 Andrology Unit, Department of Clinical Physiopathology, University of Florence, Florence, Italy; 10 Scanian Andrology Centre, Department of Urology, Malmö University Hospital, University of Lund, Lund, Sweden; 11 Department of Endocrinology, Royal Free and University College Hospital Medical School, Royal Free Hospital, Hampstead, London; 12 Department of Andrology and Reproductive Endocrinology, Medical University of Lodz, Lodz, Poland; 13 Department of Human Nutrition, University of Glasgow, Glasgow, Scotland; 14 Clinical Gerontology, The University of Manchester, Manchester Academic Health Science Centre, Hope Hospital, Salford, United Kingdom; 15 Andrology Unit, United Laboratories of Tartu University Clinics, Tartu, Estonia; 16 Department of Endocrinology, Manchester Royal Infirmary, The University of Manchester, Manchester Academic Health Science Centre, Manchester, United Kingdom; Ohio State University, United States of America

## Abstract

**Introduction:**

In this study, we aimed to investigate the association between single nucleotide polymorphisms (SNPs) within two genes involved in the NF-κB cascade (*GPR177* and *MAP3K14*) and bone mineral density (BMD) assessed at different skeletal sites, radial geometric parameters and bone turnover.

**Methods:**

Ten *GPR177* SNPs previously associated with BMD with genome-wide significance and twelve tag SNPs (r^2^≥0.8) within *MAP3K14* (±10 kb) were genotyped in 2359 men aged 40–79 years recruited from 8 centres for participation in the European Male Aging Study (EMAS). Measurement of bone turnover markers (PINP and CTX-I) in the serum and quantitative ultrasound (QUS) at the calcaneus were performed in all centres. Dual energy X-ray absorptiometry (DXA), at the lumbar spine and hip, and peripheral quantitative computed tomography (pQCT), at the distal and midshaft radius, were performed in a subsample (2 centres). Linear regression was used to test for association between the SNPs and bone measures under an additive genetic model adjusting for study centre.

**Results:**

We validated the associations between SNPs in *GPR177* and BMD_a_ previously reported and also observed evidence of pleiotrophic effects on density and geometry. Rs2772300 in *GPR177* was associated with increased total hip and LS BMD_a_, increased total and cortical vBMD at the radius and increased cortical area, thickness and stress strain index. We also found evidence of association with BMD_a_, vBMD, geometric parameters and CTX-I for SNPs in *MAP3K14*. None of the *GPR177* and *MAP3K14* SNPs were associated with calcaneal estimated BMD measured by QUS.

**Conclusion:**

Our findings suggest that SNPs in *GPR177* and *MAP3K14* involved in the NF-κB signalling pathway influence bone mineral density, geometry and turnover in a population-based cohort of middle aged and elderly men. This adds to the understanding of the role of genetic variation in this pathway in determining bone health.

## Introduction

Osteoporosis is characterized by reduced bone mass and deterioration in bone micro-architecture leading to bone fragility and increased risk of fracture [1]. It is a major health problem with the lifetime risk of fracture estimated to be about 50% and 20% at age 50 years in women and men, respectively [2]. Prospective studies suggest that there is a strong relationship between levels of bone mineral density (BMD) and subsequent risk of fracture in both men [3] and women [4], [5].

Genetic factors are important determinants of bone mass. Family and twin studies suggest that up to 80% of areal BMD (BMD_a_) at the lumbar spine (LS) and hip is determined by genetic factors [6]–[8]. Genome-wide association studies (GWAS) have identified a number of loci associated with LS and hip BMD_a_ reaching genome-wide significance (GWS), including the genes involved in the RANKL/RANK/OPG signalling pathway [9]–[12], which have also been associated with markers of bone turnover [13]. The RANKL/RANK/OPG signalling pathway has an important role in bone turnover. Interaction of RANKL (receptor activator of NF-κB ligand) with its receptor RANK (receptor activator of NF-κB) on the surface of osteoclast precursors [14] activates both canonical and non-canonical pathways of NF-κB. Subsequently, active NF-κB dimers move into the nucleus, bind to the DNA and cause gene transcription and osteoclast differentiation [15]. OPG acts as a decoy receptor for RANKL and can block its effects [14].

Rivadeneira et al. performed a meta-analysis of five GWAS of LS and femoral neck (FN) BMD_a_ in approximately 20,000 subjects [10]. They strengthened the association of *RANKL*, *RANK* and *OPG* with BMD_a_; and also identified two new loci including genes involved in the NF-κB pathway. *GPR177* is located on chromosome 1p31.3 and single nucleotide polymorphisms (SNPs) within this gene were associated with LS and FN BMD_a_ at the GWS level [10]. GPR177 which is also known as WNTLESS homolog is an activator of the NF-κB pathway [16]. It also induces osteoblast differentiation through the Wnt signalling pathway [17]. A single SNP on chromosome 17q12, rs9303521, which is located about 400 kb upstream of *MAP3K14*, was also associated with LS BMD_a_ at the GWS level in the meta-analysis [10]. *MAP3K14* encodes NIK (NF-κB inducing kinase) which induces production of active NF-κB dimers in the non-canonical pathway [15].

In this study, we aimed to validate *GPR177* SNP associations with BMD_a_ and to determine if SNPs in *MAP3K14* are associated with BMD_a_ utilizing an independent population of middle-aged and elderly men recruited in the European Male Aging Study (EMAS) [18]. We also explored the influence of *GPR177* and *MAP3K14* SNPs on bone geometry, another important determinant of bone strength. As both genes investigated here are involved in bone turnover related pathways, one would expect that their effects on bone density and geometry would, at least partly, be through altering bone turnover. Therefore, we sought to test this hypothesis. We also investigated the combined effect of strongly associated SNPs in *RANKL*, *RANK*, *OPG*, *GPR177* and *MAP3K14* on BMD_a_.

## Methods

### Ethics statement

Ethical approval for the study was obtained in accordance with local institutional requirements in each centre: Florence (Ethical Committee of the Azienda Ospedaliera Careggi & University of Florence), Leuven (Commissie Medische Ethiek UZ Gasthuisberg & KU Leuven), Lodz (Bioethical Committee of Medical University of Lodz for Human Studies), Malmö (Ethical Committee of Lund University), Manchester (North West Multi Centre Ethical Research Committee, University of Manchester & CMMCUH), Santiago de Compostela (Comité Ético de Investigación Clínica de Galicia & Universidad de Santiago de Compostela), Szeged (Human Investigation Review Board, University of Szeged) and Tartu (Ethical Committee, Medical University of Tartu). All subjects provided written informed consent. Approval for the genetic analysis described here was obtained for seven of the eight centres. Therefore, analysis was restricted to subjects from these seven centres (all centres except for Malmö, Sweden).

### Study Participants

Men aged 40–79 years were recruited from population registers in 8 European centres (Manchester, UK; Leuven, Belgium; Tartu, Estonia; Lodz, Poland; Szeged, Hungary; Florence, Italy; Santiago de Compostela, Spain; Malmö, Sweden) into the European Male Ageing Study, for further details see Lee at al. (2008) [19]. Blood samples were collected for genetic analysis. Quantitative ultrasound (QUS) at the calcaneus was performed in subjects at all centres. Dual energy X-ray absorptiometry (DXA) and peripheral quantitative computed tomography (pQCT) were performed in a subsample of subjects in two centres (Manchester, UK and Leuven, Belgium). Participants were excluded from this analysis if they reported that at least one of their parents or grandparents was born outside Europe or North America, or if they reported use of anti-osteoporotic medications or systemic glucocorticoids.

### Bone Assessments

#### DXA

DXA scans were performed in Manchester and Leuven centres using a DXA QDR 4500A device (Hologic, Inc, Waltham, MA, USA). BMD_a_ (g/cm^2^) was measured at LS (L1 to L4) and total hip (TH). All scans and measurements were performed by trained and experienced DXA technicians. The Hologic Spine Phantom was scanned daily to monitor the device performance and long-term stability. The precision of these measurements in the LS and TH were 0.57% and 0.56% in Leuven, and 0.97% and 0.97% in Manchester, respectively. Both devices were cross-calibrated with the European Spine Phantom [20].

#### pQCT

Peripheral QCT measurements were performed in the non-dominant radius using XCT-2000 scanners (Stratec, Pforzheim, Germany) in Manchester and Leuven centres following the manufacturer's standard quality assurance procedures. Total and trabecular vBMD (mg/mm^3^), and bone cross-sectional area (mm^2^) were measured at the distal radius (4%) (voxel size 0.4 mm). Cortical vBMD (mg/mm^3^); total, cortical and medullary area (mm^2^); cortical thickness (mm) and stress strain index (SSI) (mm^3^) were measured at the midshaft radius (50%) (voxel size 0.6 mm). The detailed methodology for these measurements has been described previously [21].

The European Forearm Phantom (EFP) was measured for cross-calibration between the two centres; 10 repeat measurements were taken in slices 1–4. The differences were less than precision error for total, trabecular and cortical vBMD, and cortical area. Therefore, no cross-calibration was performed between the two centres. The short term precision of 2 repeat measurements with repositioning were: 2.1% and 1.3% for total vBMD; 1.27% and 1.42% for trabecular vBMD; 0.77% and 0.71% for cortical vBMD; and 2.4% and 1.3% for cortical area; in Manchester (n = 22) and Leuven (n = 40), respectively.

#### QUS

BMD at the left calcaneus was estimated by QUS using the Sahara Clinical Sonometer (Hologic, Bedford, MA, USA) in all centres following a standardized protocol. Outputs included broadband ultrasound attenuation (BUA) (dB/MHz) and speed of sound (SOS) (m/s). Calcaneal eBMD was calculated (g/cm^2^) using the following formula: BMD = 0.002592×(BUA+SOS)−3.687. The CVs were 2.8%, 0.3% and 3.4% for BUA, SOS and BMD, respectively as described previously [13].

#### Bone Turnover Markers

Serum N-terminal propeptide of type I procollagen (PINP) (ng/ml) and C-terminal cross-linked telopeptide of type I collagen (CTX-I) (ng/ml) levels were measured in Leuven, using electrochemiluminescence immunoassay (ECLIA) (Roche Diagnostics) on samples from all centres. The detection limit of PINP assay was <5 ng/ml, and the intra-assay and inter-assay coefficients of variations (CVs) were 0.8–2.9 and 1.8–2.9%, respectively. The detection limit of CTX-I assay was 0.01 ng/ml, and the intra-assay and inter-assay CVs were 2.0–5.5% and 1.0–4.6%, respectively.

### SNPs Selection

#### GPR177

The SNPs within *GPR177* associated with LS or FN BMD_a_ at the genome-wide significant level (p<5×10^−8^) in a previous GWAS meta-analysis [10] were selected for genotyping. Where SNPs were in high linkage disequilibrium (LD) (r^2^≥0.8) based on HapMap Phase II CEPH SNP data (http://www.hapmap.org), only one of them was genotyped.

#### MAP3K14

Pair-wise tag SNPs (r^2^≥0.8) were selected for SNPs in HapMap Phase II CEPH SNP data (http://www.hapmap.org) with a minor allele frequency (MAF) of greater than 5% within *MAP3K14* and its 10 kb flanking regions using Tagger implemented in Haploview 4.0 [22].

### Genotyping and Quality Control

SNPs were genotyped using SEQUENOM MassARRAY technology following the manufacturer's instructions (http://www.sequenom.com). Sample and assay quality control thresholds were set to 90%. The SNPs deviating from Hardy-Weinberg equilibrium (HWE), p≤0.05, were excluded from the analysis.

### Statistical Analysis

The outcome variables were standardised, z = (x−μ)/σ where x = raw value of bone measure, μ = mean of bone measure and σ = standard deviation of bone measure.

The association between the SNPs and the standardised outcome variables (DXA: LS and TH BMD_a_, pQCT: radius vBMD and geometric parameters, QUS: calcaneal BMD, and bone turnover markers: PINP and CTX-I) was tested using linear regression under an additive genetic model using PLINK (1.05) [23]. The results were adjusted for study centre and also for study centre, age, height and weight. The results are presented as a percentage change (β) in a standard deviation (SD) with 95% confidence intervals for each copy of the minor allele. For significantly associated SNPs, the interaction between the SNP and centre was tested to see if the relationship between the SNP and the outcome differed across centres.

Pairs of SNPs associated with BMD_a_ and located in different genes (*RANKL*, *RANK*, *OPG*, *GPR177* and *MAP3K14*) at the same skeletal site with a p-value<0.01 were tested to determine if carrying the risk allele (allele associated with lower BMD) for both SNPs was associated with a greater effect on BMD_a_. For *RANKL*, *RANK* and *OPG*, data from previous analysis in the EMAS cohort was used [13]. In order to determine the combined effect of the two SNPs, a new combined genotype variable was defined based on the number of risk alleles each individual carried for either SNP. This new variable could have a value between 0 to 4; 0: having no copy of risk alleles for both SNPs, 1: having one copy of risk alleles for either SNPs, 2: having two copies of risk alleles for either SNPs, 3: having two copies of risk allele for one SNP and one copy of risk allele for the other one, and 4: having 2 copies of risk alleles for both SNPs. Subsequently, linear regression was used for testing the association between BMD_a_ and this new variable adjusting for centre in STATA (9.2).

## Results

### Subject Characteristics

Of the 2658 men who consented to participate in the genetic analysis, 299 were excluded: 180 failed sample quality control, 21 reported at least one of their parents or grandparents was born outside Europe or North America, and 98 reported use of anti-osteoporotic medications or systemic glucocorticoids. In total, 2359 men, mean (±SD) age 60±11 years, were included in the analysis. Bone turnover markers and ultrasound BMD were measured in almost all men (N = 2244 and 2314, respectively). DXA and pQCT were performed in a subsample of men from 2 centres (N = 588 and 560, respectively). Mean values of the bone turnover markers, QUS, DXA and pQCT parameters are presented in Table 1.

**Table 1:** Mean (SD) of bone turnover markers, DXA, pQCT and QUS parameters.

	N	Mean	SD
**Bone turnover markers**			
PINP (ng/ml)	2244	42.91	21.72
CTX-I (ng/ml)	2242	357.72	181.37
**DXA BMD_a_**			
Lumbar spine (g/cm^2^)	588	1.06	0.18
Total hip (g/cm^2^)	587	1.02	0.14
Femoral neck (g/cm^2^)	587	0.81	0.13
**pQCT**			
**4% radius**			
Total vBMD (mg/mm^3^)	560	400.66	71.21
Trabecular vBMD (mg/mm^3^)	560	203.82	42.72
Cross-sectional area (mm^2^)	560	374.62	66.31
**50% site**			
Cortical vBMD (mg/mm^3^)	560	1214.47	29.68
Cortical thickness (mm)	560	3.24	0.42
Total area (mm^2^)	560	149.31	20.72
Cortical area (mm^2^)	560	106.44	13.63
Medullary area (mm^2^)	560	42.87	15.71
SSI (mm^3^)	560	337.56	63.87
**QUS**			
BMD at calcaneus (g/cm^2^)	2314	0.54	0.14

PINP: N-terminal propeptide of type I procollagen, CTX-I: C-terminal cross-linked telopeptide of type I collagen, DXA: dual energy X-ray absorptiometry, BMD_a_: areal bone mineral density, pQCT: peripheral quantitative computed tomography, vBMD: volumetric bone mineral density, SSI: stress strain index, QUS: quantitative ultrasound.

### Genotyping

#### GPR177

Thirteen SNPs within *GPR177* were selected for genotyping. Of these, 10 SNPs were successfully genotyped and passed quality control. Rs919540, rs994082 and rs2772304 failed genotyping.

#### MAP3K14

Forty-three SNPs with a MAF>0.05 were identified in *MAP3K14* and its 10 kb flanking regions. These SNPs were tagged by 13 SNPs. Of these, 12 SNPs were successfully genotyped and passed quality control covering 98% of the selected SNPs with a MAF of more than 5% in the region. Rs2867316 failed genotyping.

### Genetic Association Analysis

For the bone turnover markers and ultrasound BMD, with α = 0.05, there was greater than 80% power to detect differences of 0.2 SD for SNPs with a MAF = 0.05 and 0.1 SD for SNPs with a MAF = 0.45 under an additive genetic model. For DXA and pQCT outcomes, with α = 0.05, there was greater than 80% power to detect differences of 0.4 SD for SNPs with a MAF = 0.05 and 0.2 SD for SNPs with a MAF = 0.45 under an additive genetic model. Statistical power was calculated using Quanto v1.2.3 software [24].

#### GPR177

**Figure 1:**
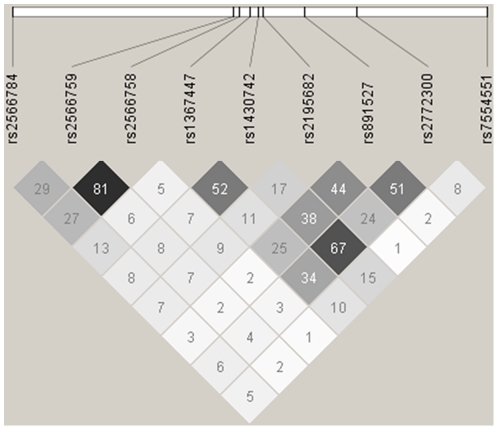
The pair-wise LD between *GPR177* SNPs associated with bone health parameters. (black r^2^ = 1, white r^2^ = 0).

Of the 10 SNPs successfully genotyped, 6 were associated with total hip BMD_a_. Rs1430742, which was the most significantly associated SNP in this locus in a GWAS meta-analysis of LS and FN BMD_a_ [10], showed the largest effect with each allele resulting in a 0.18 SD (95%CI 0.04, 0.33) p = 0.015 increase in total hip BMD_a_. Of these 6 SNPs, 2 (rs2772300 and rs7554551) also showed a significant association with LS BMD_a_ and 2 showed a non-significant effect in the same direction as total hip BMD_a_ but of a lesser magnitude (Table 2). Three of the SNPs, associated with total hip BMD_a_, also showed a significant association with total vBMD at the distal radius, with rs1430742 again showing the largest effect (0.15 SD (95%CI 0.02, 0.28) p = 0.024 per allele). rs2772300, which is in moderate LD with rs1430742 (r^2^ = 0.67) (Figure 1) was also significantly associated with increased cortical vBMD at the mid-shaft radius (0.18 SD (0.05, 0.32) p = 0.007 per allele). Other SNPs showed a change in cortical vBMD but did not attain statistical significance. Rs891257 was also significantly associated with increased cortical vBMD, but was not associated with LS or TH BMD_a_ or total vBMD (Table 3). There was no association between any of the SNPs and trabecular vBMD. The results were broadly similar when applying further adjustment for age, height and weight (Tables 2 and 3).

**Table 2:** Genetic association between *GPR177* SNPs and BMD_a_.

			Total Hip BMD_a_				LS BMD_a_			
SNP	Alleles	MAF	β(SD) (95% CI)^a^	p^a^	β(SD) (95% CI)^a^	p^b^	β(SD) (95% CI)^a^	p^a^	β(SD) (95% CI)^a^	p^b^
rs2566784	G>T	0.25	0.13 (−0.01, 0.26)	0.063	0.18 (0.06, 0.29)	0.003	0.11 (−0.01, 0.24)	0.077	0.14 (0.03, 0.26)	0.018
rs3762371	G>A	0.4	−0.07 (−0.19, 0.05)	0.261	−0.03 (−0.14, 0.07)	0.565	−0.02 (−0.14, 0.09)	0.676	−0.02 (−0.12, 0.09)	0.74
rs2566759	A>G	0.5	−0.12 (−0.24, 0.00)	0.049	−0.09 (−0.20, 0.01)	0.088	−0.04 (−0.15, 0.07)	0.495	−0.04 (−0.15, 0.06)	0.439
rs2566758	T>C	0.46	−0.13 (−0.24, −0.01)	0.039	−0.10 (−0.21, 0.00)	0.053	−0.06 (−0.17, 0.06)	0.316	−0.07 (−0.18, 0.03)	0.187
rs1367447	C>T	0.19	0.15 (−0.01, 0.30)	0.062	0.09 (−0.05, 0.23)	0.208	0.07 (−0.08, 0.22)	0.377	0.04 (−0.10, 0.18)	0.567
rs1430742	T>C	0.23	0.18 (0.04, 0.33)	0.015	0.17 (0.04, 0.30)	0.011	0.13 (−0.01, 0.27)	0.077	0.14 (0.01, 0.27)	0.036
rs2195682	C>T	0.39	−0.16 (−0.28, −0.04)	0.007	−0.14 (−0.24, −0.03)	0.011	−0.11 (−0.22, 0.01)	0.063	−0.11 (−0.21, 0.00)	0.052
rs891527	A>T	0.42	0.05 (−0.07, 0.17)	0.411	0.06 (−0.04, 0.17)	0.242	0.02 (−0.10, 0.13)	0.751	0.04 (−0.07, 0.15)	0.446
rs2772300	G>A	0.28	0.15 (0.02, 0.29)	0.026	0.18 (0.06, 0.30)	0.003	0.16 (0.03, 0.29)	0.013	0.20 (0.08, 0.32)	0.001
rs7554551	T>C	0.33	0.17 (0.05, 0.30)	0.005	0.19 (0.08, 0.29)	0.001	0.14 (0.03, 0.26)	0.016	0.17 (0.06, 0.28)	0.003

a adjusted for study centre;

b adjusted for study centre, age, height and weight.

**Table 3:** Genetic association between *GPR177* SNPs and BMD_v_.

			Total vBMD (4%)				Cortical vBMD (50%)			
SNP	Alleles	MAF	β(SD) (95% CI)^a^	p^a^	β(SD) (95% CI)^a^	p^b^	β(SD) (95% CI)^a^	p^a^	β(SD) (95% CI)^a^	p^b^
rs2566784	G>T	0.25	0.08 (−0.04, 0.19)	0.178	0.09 (−0.02, 0.20)	0.099	0.04 (−0.09, 0.18)	0.52	0.06 (−0.07, 0.19)	0.365
rs3762371	G>A	0.40	−0.02 (−0.13, 0.08)	0.639	0.01 (−0.09, 0.11)	0.887	−0.03 (−0.15, 0.09)	0.641	−0.01 (−0.12, 0.11)	0.904
rs2566759	A>G	0.50	−0.05 (−0.16, 0.05)	0.308	−0.02 (−0.12, 0.08)	0.664	−0.08 (−0.20, 0.04)	0.17	−0.06 (−0.17, 0.06)	0.331
rs2566758	T>C	0.46	−0.10 (−0.21, 0.00)	0.053	−0.06 (−0.16, 0.04)	0.222	−0.09 (−0.21, 0.03)	0.133	−0.06 (−0.18, 0.05)	0.291
rs1367447	C>T	0.19	0.12 (−0.02, 0.25)	0.093	0.09 (−0.04, 0.22)	0.196	0.11 (−0.05, 0.27)	0.170	0.07 (−0.08, 0.23)	0.362
rs1430742	T>C	0.23	0.15 (0.02, 0.28)	0.024	0.12 (0.00, 0.25)	0.057	0.11 (−0.04, 0.26)	0.158	0.06 (−0.09, 0.21)	0.427
rs2195682	C>T	0.39	−0.10 (−0.21, 0.00)	0.053	−0.07 (−0.17, 0.03)	0.164	−0.10 (−0.22, 0.02)	0.094	−0.10 (−0.21, 0.02)	0.108
rs891527	A>T	0.42	0.04 (−0.07, 0.14)	0.504	0.02 (−0.08, 0.12)	0.72	0.16 (0.04, 0.28)	0.011	0.14 (0.02, 0.25)	0.023
rs2772300	G>A	0.28	0.13 (0.01, 0.23)	0.036	0.10 (−0.01, 0.22)	0.074	0.18 (0.05, 0.32)	0.007	0.15 (0.01, 0.28)	0.031
rs7554551	T>C	0.33	0.12 (0.01, 0.23)	0.028	0.10 (0.00, 0.21)	0.047	0.03 (−0.09, 0.15)	0.641	0.01 (−0.11, 0.13)	0.914

a adjusted for study centre;

b adjusted for study centre, age, height and weight.

SNPs in *GPR177* which were associated with BMD also showed evidence of association with geometric parameters of bone (Table 4). Two SNPs (rs2566759 and rs2195682 were associated with cross-sectional area at the distal radius. Rs277230 and rs1430742 were associated with increased cortical area at the mid-shaft radius and rs2772300 was also associated with increased cortical thickness and SSI. Rs7554551 was associated with cortical thickness at the midshaft radius. There was no association between SNPs in *GPR177* and bone turnover markers or ultrasound eBMD.

**Table 4:** *GPR177* SNP associations with radius geometric parameters.

SNP	Alleles	MAF	Phenotype	Skeletal Site	β(SD) (95% CI)^a^	p^a^	β(SD) (95% CI)^b^	p^b^
rs2566759	A>G	0.5	Cross-sectional area	4% radius	0.11 (0.01, 0.21)	0.037	0.10 (0.00, 0.20)	0.045
rs2566758	T>C	0.46	Cross-sectional area	4% radius	0.11 (0.01, 0.22)	0.030	0.09 (0.00, 0.19)	0.060
rs1430742	T>C	0.23	Cortical area	50% radius	0.17 (0.02, 0.32)	0.031	0.14 (0.00, 0.27)	0.053
rs2772300	G>A	0.28	Cortical area	50% radius	0.17 (0.03, 0.30)	0.015	0.18 (0.05, 0.30)	0.005
			Cortical Thickness	50% radius	0.17 (0.04, 0.31)	0.012	0.17 (0.04, 0.30)	0.011
			SSI	50% radius	0.15 (0.01, 0.28)	0.030	0.16 (0.03, 0.28)	0.013
rs7554551	T>C	0.33	Cortical Thickness	50% radius	0.15 (0.03, 0.27)	0.018	0.15 (0.03, 0.27)	0.015

SSI: stress strain index;

a adjusted for study centre;

b adjusted for study centre, age, height and weight.

#### MAP3K14

Two SNPs showed evidence of association with BMD. Rs8065345 was significantly associated with a 0.25 SD (95%CI 0.10, 0.41) p = 0.002 per allele increase in LS BMD_a_ and was also associated with increased cortical area and cortical thickness. Rs7215764 was significantly associated with increased total hip BMD_a_ and cortical vBMD at the mid-shaft radius. Three further SNPs were associated with geometric parameters, rs11651968 was associated with decreased cortical area at the mid-shaft radius and rs1785379 was associated with decreased medullary area and increased cortical thickness at the mid-shaft radius. Rs16939948 was associated with total area at the mid-shaft radius; however, this association became non-significant after further adjustment for age, height and weight (Table 5). Further adjusting the associations between *MAP3K14* SNPs and bone health parameters for age, height and weight gave broadly similar results with effect estimates of similar magnitude. LD between the associated SNPs was weak, r^2^≤0.13. The results for all *MAP3K14* SNPs with LS and total hip BMD_a_ and total and cortical vBMD are given in Tables S1 & S2 respectively.

**Table 5:** *MAP3K14* SNP associations with bone mineral density and radius geometric parameters.

SNP	Alleles	MAF	Location	Phenotype	Skeletal Site	β(SD) (95% CI)^a^	p^a^	β(SD) (95% CI)^b^	p^b^
rs8065345	A>G	0.15	3′ downstream	BMD_a_	Lumbar spine	0.25 (0.10, 0.41)	0.002	0.21 (0.06, 0.35)	0.006
				Cortical area	50% radius	0.21 (0.05, 0.38)	0.011	0.16 (0.01, 0.31)	0.041
				Cortical thickness	50% radius	0.24 (0.07, 0.40)	0.004	0.22 (0.06, 0.38)	0.006
rs11651968	C>T	0.48	3′ downstream	Cortical area	50% radius	−0.13 (−0.25, −0.01)	0.031	−0.11, −0.22, 0.01)	0.062
rs7215764	C>G	0.25	3′ downstream	BMD_a_	Total hip	0.17 (0.03, 0.31)	0.021	0.10 (−0.03, 0.22)	0.136
				Cortical vBMD	50% radius	0.17 (0.03, 0.32)	0.020	0.17 (0.03, 0.31)	0.016
rs17685379	C>G	0.14	Intronic	Medullary area	50% radius	−0.17 (−0.35, 0.00)	0.048	−0.19 (−0.36, −0.02)	0.031
				Cortical thickness	50% radius	0.18 (0.01, 0.35)	0.043	0.22 (0.05, 0.38)	0.011
rs16939948	T>C	0.05	Intronic	Total area	50% radius	0.29 (0.01, 0.57)	0.044	0.23 (−0.03, 0.50)	0.089

BMD_a_: areal bone mineral density, vBMD: volumetric bone mineral density;

a adjusted for study centre;

b adjusted for study centre, age, height and weight.

In contrast to *GPR177*, there were three *MAP3K14* SNPs associated with CTX-I serum levels; rs4792847 (0.07 SD (95%CI 0.01, 0.12) p = 0.013), rs4792849 (0.06 SD (95%CI 0.00, 0.12) p = 0.042) and rs4328483 (0.08 SD (95%CI 0.03, 0.14) p = 0.003). None of the SNPs were associated with PINP serum levels. There was a moderate LD between these three SNPs (0.69<r^2^>0.39). There was no association between SNPs in *MAP3K14* SNPs and ultrasound BMD.

### The combined effect of BMD associated SNPs in different pathway genes

**Figure 2:**
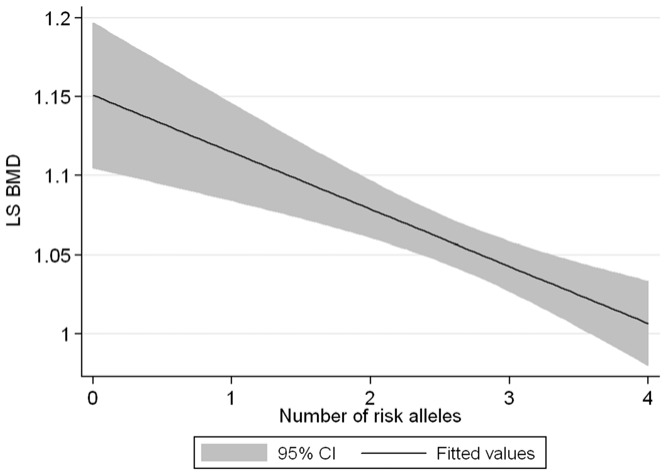
The decline in lumbar spine areal BMD by increasing number of carried risk alleles of rs9594738 (*RANKL*) and rs8065345 (*MAP3K14*).

A single pair of SNPs, rs9594738 in *RANKL* and rs8065345 in *MAP3K14*, which were both associated with LS BMD_a_ (p = 0.001) met the inclusion criteria for examining their combined effect. Five hundred seventy-eight subjects were included in the analysis: 5, 47, 188, 250 and 88 subjects had 0 to 4 copies of the risk alleles for either rs9594738 in *RANKL* or rs8065345 in *MAP3K14*, respectively. LS BMD_a_ was reduced by 0.04 g/cm^2^ (95% CI −0.06, −0.02) per risk alleles carried (p = 4.6×10^−6^) (Figure 2).

## Discussion

In this study, we investigated the association between SNPs within two genes involved in the NF-κB cascade (*GPR177* and *MAP3K14*) and bone mineral density assessed at different skeletal sites, radial geometric parameters and bone turnover. Additionally, for the first time, we investigated the potential combined effect of two associated SNPs in *MAP3K14* and *RANKL* (previously reported) on LS BMD_a_.

First, we attempted to validate the association between ten SNPs in *GPR177* and BMD_a_ reported in a GWAS meta-analysis [10], six of which showed significant association with total hip BMD_a_ in the same direction in EMAS. Two of these SNPs were also significantly associated with LS BMD_a_ and another 2 showed suggestive association (p<0.1) with LS BMD_a_. This weaker evidence of association with LS BMD_a_ may have arisen due to the presence of concomitant disease such as osteoarthritis which can artificially raise BMD of the spine in an elderly cohort, making it harder to detect an association, particularly in a cohort of modest sample size. The results confirm the GWAS meta-analysis findings and show that SNPs in *GPR177* influence BMD_a_ at osteoporotic sites in a population of middle aged and elderly men at that their effects can be detected in a relatively modest sample size.

In addition, SNPs in *GPR177* were also associated with total and cortical BMD at the radius, which is a novel finding, however despite testing in a larger sample size, there was no evidence of association with calcaneal eBMD as measured by ultrasound, a pattern which has been observed for other BMD susceptibility genes [13]. The findings reported here also suggest that these SNPs may have pleiotrophic effects on bone as SNPs associating with cortical vBMD were also associated with geometric parameters at the radius. Rs2772300, which was associated with increased BMD_a_ at the hip and LS and total and cortical vBMD at the radius, was also associated with increased cortical area, thickness and stress strain index. It seems unlikely that these effects are mediated by bone turnover as no association was observed between these SNPs and the bone turnover markers CTX-I and PINP, in a larger sample size. Variation in the level of markers may be one explanation for this. We attempted to reduce variability in the bone turnover markers by taking fasting blood samples and measuring them in a single laboratory (to reduce technical variability). Further, the bone turnover markers selected here (PINP and CTX-I) both had low intra-assay and inter-assay CVs minimizing their analytical variability. Turnover marker levels, however, reflect current skeletal turnover and may not necessarily reflect previous levels, such misclassification may in part explain the absence of any observed significant associations.

We also looked at the association of common SNPs (MAF≥0.05) in *MAP3K14* with bone health parameters as this gene was previously highlighted as a good candidate, however there is no LD between the SNPs tested here in the *MAP3K14* gene and the 17q12 SNP associated with BMD_a_ [10]. There was evidence of association between *MAP3K14* SNPs and BMD_a_, vBMD and radius geometric parameters, however unlike *GPR177*, there were no SNPs showing association with vBMD and geometric parameters, therefore the findings should be interpreted with caution. This is the first time that genetic variation in the *MAP3K14* gene has been investigated with bone health parameters and in a modest sample size, therefore replication of these findings is required in larger cohorts.

Notably, we also found that individuals carrying risk alleles for SNPs in the *MAP3K14* and *RANKL* genes had a significantly lower LS BMD_a_ suggesting that, despite the modest effects of single SNPs, in individuals carrying multiple risk alleles for SNPs in the RANKL/RANK/OPG and Nf-κb signalling pathways they could have a profound effect on bone health. This finding also requires replication in independent cohorts.

Important potential limitations of this study are that false positive associations might have been produced due to population stratification and multiple testing. We tried to minimize the probability of population stratification by excluding subjects of non-European ancestry and did not observe any between centre heterogeneity. However, we were unable to explore population substructure using methods such as genomic control or principal component analysis as these require data on a large number of SNPs. Based on the method described by Li and Ji [25], 19 independent SNPs (*GPR177*: 8 and *MAP3K14*: 11 SNPs) were investigated in this study. The majority of the positive findings would not remain significant if Bonferroni correction (p<0.0026, 0.05/19) was applied to correct for multiple testing. On the other hand, we may have missed some genuine associations due to low statistical power. Lack of replication for association of some SNPs with BMD_a_ may also be due to the relatively small number of subjects in our DXA subsample compared with the GWAS meta-analysis. In addition, EMAS is a male population whereas the GWAS meta-analysis data was based on studies of both men and women.

Assessment of cortical vBMD using pQCT is subject to the partial volume effect; the thinner the cortices the lower vBMD. In our analysis, we used a higher threshold (960 mg/cm^3^) for analysis of cortical vBMD rather than the traditional 710 mg/cm^3^ threshold [26] to reduce the likelihood of this effect. In addition, most of the SNPs associated with cortical vBMD were also associated with areal and/or volumetric BMD at other skeletal sites but they were not associated with cortical thickness suggesting that they are real findings rather than false positive results arising due to technical artefact.

In summary, our findings suggest that SNPs within *GPR177* and *MAP3K14* involved in the NF-κB pathway influence BMD at both axial (LS and Hip) and peripheral sites (radius) and they also influence radius geometry. We also found some evidence of association between SNPs within *MAP3K14* and bone turnover. However, these findings require replication in independent populations. If confirmed, fine mapping and functional studies will be needed to identify the causal variants. In addition, combinations of SNPs from different genes in the NF-κB and RANKL/RANK/OPG pathways may have substantial effects on individuals carrying risk alleles of both SNPs highlighting the importance of these pathways in the genetic basis of bone health.

## Supporting Information

Table S1 **Genetic association between** ***MAP3K14*** **SNPs and BMD_a_.**
(DOC)

Table S2 **Genetic association between** ***MAP3K14*** **SNPs and BMD_v_.**
(DOC)
