# Supplementary material for: Polymorphisms in Genes Involved in the NF-κB Signalling Pathway Are Associated with Bone Mineral Density, Geometry and Turnover in Men
Source: PLoS One. 2011 Nov 21;6(11):e28031. doi: 10.1371/journal.pone.0028031 (PMC3221678; doi:10.1371/journal.pone.0028031)
Supplement: Table S1 — Genetic association between MAP3K14 SNPs and BMDa. (DOC) [file pone.0028031.s001.doc]

**Table S1. Genetic association between *MAP3K14* SNPs and BMDa**

| **SNP** | **Alleles** | **MAF** | **Total Hip BMD_a_** | | | | **LS BMD_a_** | | | |
| --- | --- | --- | --- | --- | --- | --- | --- | --- | --- | --- |
|  |  |  | **β(SD) (95% CI)^a^** | **p^a^** | **β(SD) (95% CI)^b^** | **p^b^** | **β(SD) (95% CI)^a^** | **p^a^** | **β(SD) (95% CI)^b^** | **p^b^** |
| rs8065345 | A>G | 0.16 | 0.11 (-.05, 0.27) | 0.192 | 0.06 (-0.09, 0.21) | 0.415 | 0.25 (0.10, 0.41) | 0.002 | 0.21 (0.06, 0.35) | 0.006 |
| rs2291448 | A>G | 0.07 | -0.16 (-0.41, 0.09) | 0.216 | -0.02 (-0.24, 0.21) | 0.873 | -0.20 (-0.44, 0.04) | 0.102 | -0.11 (-0.34, 0.12) | 0.337 |
| rs11651968 | C>T | 0.44 | -0.05 (-0.17, 0.07) | 0.402 | -0.01 (-0.12, 0.10) | 0.862 | -0.08 (-0.20, 0.03) | 0.169 | -0.04 (-0.15, 0.06) | 0.417 |
| rs7215764 | C>G | 0.25 | 0.17 (0.03, 0.31) | 0.021 | 0.10 (-0.03, 0.22) | 0.136 | 0.12 (-0.02, 0.26) | 0.082 | 0.08 (-0.05, 0.21) | 0.231 |
| rs17685379 | C>G | 0.14 | -0.02 (-0.2, 0.15) | 0.783 | 0.01 (-0.14, 0.16) | 0.880 | 0.06 (-0.11, 0.22) | 0.487 | 0.06 (-0.09, 0.22) | 0.425 |
| rs16939948 | T>C | 0.05 | -0.28 (-0.56, 0.01) | 0.059 | -0.31 (-0.56, -0.06) | 0.015 | 0.02 (-0.25, 0.29) | 0.894 | 0.00 (-0.25, 0.26) | 0.972 |
| rs2074292 | A>G | 0.47 | 0.03 (-0.10, 0.15) | 0.676 | 0.00 (-0.11, 0.10) | 0.973 | 0.08 (-0.03, 0.20) | 0.152 | 0.07 (-0.04, 0.18) | 0.216 |
| rs4792847 | G>A | 0.50 | -0.03 (-0.15, 0.09) | 0.590 | -0.01 (-0.12, 0.09) | 0.786 | 0.04 (-0.08, 0.15) | 0.546 | 0.05 (-0.06, 0.15) | 0.390 |
| rs17686001 | G>A | 0.19 | -0.03 (-0.17, 0.11) | 0.671 | -0.02 (-0.15, 0.10) | 0.709 | -0.05 (-0.18, 0.09) | 0.492 | -0.05 (-0.17, 0.08) | 0.455 |
| rs4792849 | G>A | 0.28 | 0.04 (-0.09, 0.17) | 0.573 | 0.05 (-0.07, 0.16) | 0.441 | 0.00 (-0.13, 0.12) | 0.976 | 0.02 (-0.10, 0.13) | 0.784 |
| rs4328483 | G>T | 0.43 | -0.01 (-0.13, 0.11) | 0.880 | 0.02 (-0.09, 0.13) | 0.735 | 0.02 (-0.09, 0.14) | 0.712 | 0.04 (-0.07, 0.15) | 0.475 |

MAF: Minor allele frequency; BMD_a_: Areal bone mineral density; ^a^ adjusted for study centre; ^b^ adjusted for study centre, age, height and weight
