# Supplementary material for: Polymorphisms in Genes Involved in the NF-κB Signalling Pathway Are Associated with Bone Mineral Density, Geometry and Turnover in Men
Source: PLoS One. 2011 Nov 21;6(11):e28031. doi: 10.1371/journal.pone.0028031 (PMC3221678; doi:10.1371/journal.pone.0028031)
Supplement: Table S2 — Genetic association between MAP3K14 SNPs and BMDv. (DOC) [file pone.0028031.s002.doc]

**Table S2. Genetic association between *MAP3K14* SNPs and BMD**v

| **SNP** | **Alleles** | **MAF** | **Total vBMD (4%)** | | | | **Cortical vBMD (50%)** | | | |
| --- | --- | --- | --- | --- | --- | --- | --- | --- | --- | --- |
|  |  |  | **β(SD) (95% CI)^a^** | **p^a^** | **β(SD) (95% CI)^b^** | **p^b^** | **β(SD) (95% CI)^a^** | **p^a^** | **β(SD) (95% CI)^b^** | **p^b^** |
| rs8065345 | A>G | 0.16 | 0.13 (-0.01, 0.27) | 0.078 | 0.14 (0.00, 0.28) | 0.044 | 0.14 (-0.03, 0.30) | 0.104 | -0.07 (-0.19, 0.05) | 0.256 |
| rs2291448 | A>G | 0.07 | -0.18 (-0.40, 0.04) | 0.105 | -0.14 (-0.35, 0.07) | 0.188 | -0.16 (-0.41, 0.09) | 0.204 | -0.11 (-0.39, 0.16) | 0.418 |
| rs11651968 | C>T | 0.44 | -0.04 (-0.15, 0.06) | 0.405 | -0.05 (-0.15, 0.05) | 0.327 | -0.06 (-0.18, 0.06) | 0.337 | 0.04 (-0.13, 0.21) | 0.658 |
| rs7215764 | C>G | 0.25 | 0.06 (-0.06, 0.19) | 0.327 | 0.04 (-0.08, 0.16) | 0.533 | 0.17 (0.03, 0.32) | 0.020 | -0.14 (-0.27, 0.00) | 0.051 |
| rs17685379 | C>G | 0.14 | -0.05 (-0.20, 0.10) | 0.519 | -0.02 (-0.16, 0.13) | 0.804 | 0.02 (-0.15, 0.19) | 0.815 | 0.09 (-0.03, 0.21) | 0.128 |
| rs16939948 | T>C | 0.05 | 0.11 (-0.14, 0.35) | 0.403 | 0.10 (-0.13, 0.34) | 0.396 | -0.07 (-0.35, 0.21) | 0.634 | -0.14 (-0.38, 0.11) | 0.266 |
| rs2074292 | A>G | 0.47 | 0.06 (-0.05, 0.16) | 0.307 | 0.05 (-0.05, 0.15) | 0.358 | 0.09 (-0.03, 0.22) | 0.130 | 0.00 (-0.11, 0.12) | 0.935 |
| rs4792847 | G>A | 0.50 | 0.01 (-0.09, 0.12) | 0.811 | 0.02 (-0.08, 0.12) | 0.717 | 0.00 (-0.12, 0.12) | 0.951 | 0.01 (-0.11, 0.12) | 0.915 |
| rs17686001 | G>A | 0.19 | -0.05 (-0.17, 0.07) | 0.422 | -0.04 (-0.16, 0.08) | 0.504 | -0.14 (-0.28, 0.00) | 0.056 | 0.01 (-0.11, 0.14) | 0.834 |
| rs4792849 | G>A | 0.28 | 0.04 (-0.07, 0.15) | 0.489 | 0.02 (-0.08, 0.13) | 0.672 | 0.02 (-0.11, 0.15) | 0.794 | 0.17 (0.03, 0.31) | 0.016 |
| rs4328483 | G>T | 0.43 | -0.03 (-0.13, 0.08) | 0.599 | -0.02 (-0.13, 0.08) | 0.629 | -0.01 (-0.13, 0.11) | 0.910 | 0.13 (-0.04, 0.29) | 0.127 |

MAF: Minor allele frequency; vBMD: Volumetric bone mineral density; ^a^ adjusted for study centre; ^b^ adjusted for study centre, age, height and weight
